# Chronic Conditions and Loneliness Among Nursing Home Residents: An Analysis of the Health & Retirement Study Wave 15

**DOI:** 10.1093/geroni/igaf122.3108

**Published:** 2025-12-31

**Authors:** LaTonya Smith, Laurie Theeke

**Affiliations:** The George Washington University, Westfield, New Jersey, United States; The George Washington University, Washington, District of Columbia, United States

## Abstract

Approximately 22-42% of nursing home residents report loneliness due to limited social interactions, loss of autonomy, and deteriorating health. This examines the relationships among loneliness and chronic physical and psychological conditions in nursing home residents using data from the Health and Retirement Study (HRS) Wave 15 (2020). Data from 131 nursing home residents were analyzed to explore associations between loneliness and chronic conditions, while considering demographics of age, gender, education, income, and marital status. Descriptive and bivariate statistics were used. The median number of nights in the nursing home was 609. The mean age of participants was 81.7 years (SD = 10.78), 69.5% female, majority reported no current income. 74.4% were high school graduates or higher, and the majority (55%) were widowed. Feeling lonely was prevalent (37.7%), 22.6% felt depressed and 34% rated their memory poor. Functional impairment was evident with 33.3% struggling with instrumental ADLs, reporting difficulty with walking (64.6%), dressing (62%), eating (33.1%), toileting (56.8%) in/out of bed (51.2%). Loneliness correlated with depressive symptoms (r = .509, p < .001). Psychological problems were related to high blood pressure (r = .920, p < .001), stroke (r = .942, p < .001), and diabetes (r = .940, p < .001), highlighting the interconnectedness of mental and physical health conditions. Residents with lung disease felt more loneliness than without (X2 =4.418, p=.036). The findings of this study highlight interplay between loneliness and physical and psychological conditions for those in long-term care, making it critical to address loneliness in this setting.

